# Antimicrobial LDPE/EVOH Layered Films Containing Carvacrol Fabricated by Multiplication Extrusion

**DOI:** 10.3390/polym10080864

**Published:** 2018-08-04

**Authors:** Max Krepker, Cong Zhang, Nadav Nitzan, Ofer Prinz-Setter, Naama Massad-Ivanir, Andrew Olah, Eric Baer, Ester Segal

**Affiliations:** 1 Department of Biotechnology and Food Engineering, Technion-Israel Institute of Technology, Haifa 3200003, Israel; makskrepker@gmail.com (M.K.); nitzannadav@technion.ac.il (N.N.); oferp@campus.technion.ac.il (O.P.-S.); naamam@bfe.technion.ac.il (N.M.-I.); 2 Center for Layered Polymeric Systems, Department of Macromolecular Science and Engineering, Case Western Reserve University, Cleveland, OH 44106-7202, USA; cxz177@case.edu (C.Z.); amo5@case.edu (A.O.); exb6@case.edu (E.B.); 3 The Russell Berrie Nanotechnology Institute, Technion-Israel Institute of Technology, Haifa 3200003, Israel

**Keywords:** antimicrobial, EVOH, essential oils, carvacrol, halloysite nanotubes, multilayered films

## Abstract

This work describes the fabrication of antimicrobial multilayered polymeric films containing carvacrol (used as a model essential oil) by co-extrusion and multiplication technique. The microlayering process was utilized to produce films, with up to 65 alternating layers, of carvacrol-containing low-density polyethylene (LDPE) and ethylene vinyl alcohol copolymer (EVOH). Carvacrol was melt compounded with LDPE or loaded into halloysite nanotubes (HNTs) in a pre-compounding step prior film production. The detailed nanostructure and composition (in terms of carvacrol content) of the films were characterized and correlated to their barrier properties, carvacrol release rate, and antibacterial and antifungal activity. The resulting films exhibit high carvacrol content despite the harsh processing conditions (temperature of 200 °C and long processing time), regardless of the number of layers or the presence of HNTs. The multilayered films exhibit superior oxygen transmission rates and carvacrol diffusivity values that are more than two orders of magnitude lower in comparison to single-layered carvacrol-containing films (i.e., LDPE/carvacrol and LDPE/(HNTs/carvacrol)) produced by conventional cast extrusion. The (LDPE/carvacrol)/EVOH and (LDPE/[HNTs/carvacrol])/EVOH films demonstrated excellent antimicrobial efficacy against *E. coli* and *Alternaria alternata* in *in vitro* micro-atmosphere assays and against *A. alternata* and *Rhizopus* in cherry tomatoes, used as the food model. The results presented here suggest that sensitive essential oils, such as carvacrol, can be incorporated into plastic polymers constructed of tailored multiple layers, without losing their antimicrobial efficacy.

## 1. Introduction

Polymeric materials with antimicrobial activity have gained significant interest over the past decade. These polymers hold immense potential in combating bacterial and fungal contaminations, including those involving pathogenic microorganisms [1,2,3]. Among these polymeric systems, biocide-releasing polymers, in which molecules or nanoparticles with biocidical activity are released from the polymer matrix, have been increasingly studied [4,5]. However, potential health and safety risks associated with the release of synthetic antimicrobials (e.g., antibiotics) and nanoparticles could limit the application of these materials [2]. Essential oils (EOs), which are natural and highly-effective antimicrobials against both bacteria and fungi [6], are categorized as GRAS (generally recognized as safe) by the Food and Drug Administration (FDA). Numerous studies have highlighted the immense potential of these antimicrobial compounds in food packaging, pharmaceuticals and hygiene [7,8,9,10]. The incorporation of EOs into polymers offers significant advantages in terms of their broad spectrum activity, safety, and as they can be released as a vapor, their antimicrobial function does not require direct contact with the target microorganism [10,11,12,13,14]. Yet, the integration of these sensitive compounds with commodity polymers using conventional manufacturing techniques is challenging, due to their loss during high-temperature processing and diminished antimicrobial efficacy [15,16,17,18,19,20].

In our recent studies, we have demonstrated that Halloysite nanotubes (HNTs), which are naturally-occurring clays with a characteristic tubular structure and chemical composition similar to kaolin [21,22], can serve as active carriers for carvacrol or its synergistic mixture with thymol [23,24]. Entrapment of these model EOs in HNTs was demonstrated to enhance the thermal stability of these volatile compounds and allowing their high-temperature melt compounding (up to 250 °C) with various polymers, including low-density polyethylene (LDPE) [23,24], polyamide [25] and polypropylene [26]. The resulting polymer nanocomposite films exhibit high EOs content, a sustained release profile of the volatile compounds, and excellent antimicrobial properties, both in synthetic media and in complex food systems [23,24,25,26]. The capability of processing EOs at high temperatures opens new opportunities to produce more sophisticated materials with superior properties and advanced functionalities. It should be noted that several other studies have reported on polymeric films containing EOs encapsulated in HNTs [27,28,29,30,31]; however, the majority of these films were prepared by solution casting.

In this study, we investigate the feasibility of fabricating complex antimicrobial EOs-containing multilayered films by layer-multiplication coextrusion [32]. In the latter technique, coextrusion through a series of layer multiplying dies allows for producing sophisticated films containing numerous layers with a tailored structure and thickness from the micro- to the nanoscale [32,33]. Herein, we produce multilayered films (number of layers varies from 9 to 65) comprising of alternating layers of carvacrol-containing LDPE and ethylene vinyl alcohol copolymer (EVOH) by layer-multiplication coextrusion. EVOH copolymers are well known for their outstanding oxygen and odor barrier properties [34,35], including organic compounds such as EOs [36]. However, due to its polar nature, EVOH tends to adsorb moisture which in turn impedes with its barrier properties. This moisture-dependent behavior was exploited for controlling the release of EOs from EVOH coating on different polymeric substrates [37,38,39].

First, carvacrol, a model EO, was loaded into HNTs in a pre-compounding step. Next, the resulting HNTs/carvacrol hybrids were melt-compounded with LDPE, followed by a high-temperature multilayer coextrusion process in which the LDPE/(HNTs/carvacrol) compound and EVOH were coextruded to produce layered (LDPE/[HNTs/carvacrol])/EVOH films. The detailed nanostructure and composition (in terms of carvacrol content) of the films were characterized and correlated to their barrier properties, carvacrol release rate, as well as antibacterial and antifungal activity, both *in vitro* and in real food systems.

## 2. Materials and Methods 

### 2.1. Materials

Halloysite Nanotubes (HNTs) were supplied by NaturalNano (Rochester, NY, USA) and dried at 150 °C for 3 h prior to use. Low-density polyethylene (LDPE), Ipethene 320, is supplied by Carmel Olefins Ltd. (Haifa, Israel) with melt flow rate of 2 g/10 min. Carvacrol (1-methyl-4-(1-methylethylidene)cyclohexene, >97%, CAS 586-62-9), Yeast extract and Tryptone for Lysogeny broth (LB) medium, NaCl, Bacto agar, Nutrient Broth (NB) medium, Potato Dextrose Agar (PDA) are purchased from Sigma Aldrich (Rehovot, Israel). NB bacto-agar is purchased from Becton Dickinson (Sparks, MD, USA).

Ethylene vinyl alcohol (EVAL E105B), (EVOH) containing 44 mole percent of ethylene was purchased from Kuraray, Tokyo Japan. The properties reported by the manufacturer of the EVOH are a density of 1.11 g cm^−3^, a melting temperature of 165 °C, a glass transition temperature of 53 °C, and an oxygen transmission rate at 20 °C and a 65% relative humidity of 0.03 cc·mm/(m^2^·day).

### 2.2. Preparation of HNTs Loaded with Carvacrol

HNTs were dried at 150 °C for 3 h prior to use to remove adsorbed moisture. HNTs/carvacrol hybrids were prepared by shear mixing of carvacrol with HNTs followed by ultrasonication (Vibra cell VCX 750 instrument, Sonics & Materials Inc., Newtown, CT, USA) at a constant amplitude of 40% for 20 min on ice [23,24,25,26]. NOTATION: This procedure should be performed in a fume hood to minimize inhalation of HNTs [40].

### 2.3. Preparation of Films

**Figure 1:**
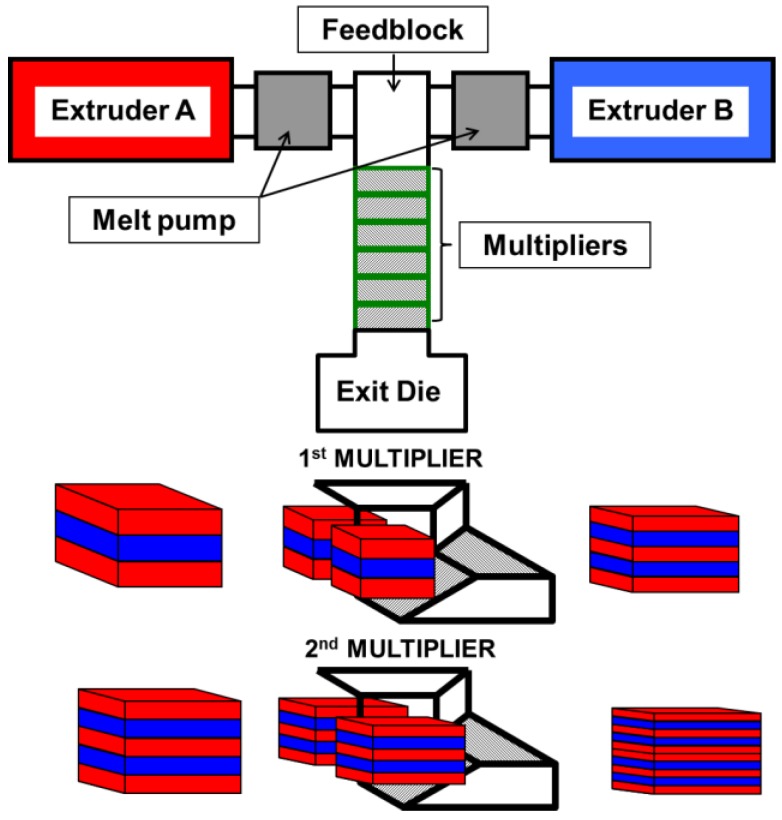
Schematic illustration of the coextrusion and multiplication line used for the fabrication of the multilayered films.

The forced-assembly multilayer coextrusion process was utilized to fabricate several different multilayer films having alternating layers of LDPE/EVOH, (LDPE/carvacrol)/EVOH, (LDPE/HNTs)/EVOH and (LDPE/[HNTs/carvacrol])/EVOH. This process, illustrated in Figure 1, comprises two single screw extruders, two melt pumps, a feed-block and a series of multipliers. During this process two coextruded polymer melts form three vertical layers within the feed-block. This three-layer melt structure then proceeds through a series of multipliers. Within each multiplier the melt is sectioned vertically and reoriented to double the number of layers as shown in Figure 1. The coextrusion temperature was selected on the rheological compatibility of the LDPE and the EVOH. The rheological properties of the polymer melts were initially determined as a function of temperature by using a Kayeness Galaxy 1 melt flow indexer (MFI) at a shear rate of 10 s^−1^ in order to simulate the flow conditions during multilayer coextrusion. This analysis identified 200 °C as the optimum multilayer coextrusion temperature. Films having 9, 17, 33 and 65 layers were produced with LDPE as the outer layers. The film thickness was maintained at a constant 100 µm (note that the actual film thickness was found to vary across the machine direction from 95 to 105 µm). The composition ratio between LDPE and EVOH within each film was kept as 80% to 20% as shown in Table 1. For comparison, LDPE was melt compounded at 150 °C with, pure carvacrol and HNTs/carvacrol hybrids, as shown in Table 1, using a 25-mm twin-screw extruder (Berstorff, Munich, Germany) with L/D ratio of 25:1 at a screw speed of 300 rpm and feeding rate of 5 kg h^−1^. Following the melt compounding process, ~100 µm thick films were prepared by cast extrusion using a 45-mm screw diameter extruder (Dr. Collin, Ebersberg, Germany) at 150 °C.

### 2.4. Characterization of Films

#### 2.4.1. High-Resolution Scanning Electron Microscopy

For cross-sectional high-resolution scanning electron microscope (HR-SEM) imaging, a polymer film was embedded in epoxy (Agar 100 resin, Agar Scientific, Stansted, UK) cured at 60 °C for 24 h. The cured epoxy block was then sectioned (along the transverse direction) at −120 °C in liquid nitrogen by a diamond knife mounted on Reichert Ultracut Ultra-microtome. The morphology of the cross-sectioned films was studied using a Carl Zeiss Ultra Plus instrument operated at 1 keV accelerating voltage.

#### 2.4.2. Thermal Gravimetric Analysis (TGA)

The post-processing content of carvacrol in the films was investigated by thermal gravimetric analysis (TGA) using a TGA-Q5000 system (TA Instruments, Newcastle, DE, USA). The films were heated under nitrogen atmosphere from room temperature to 600 °C at a heating rate of 20 °C min^−1^. The results were analyzed using Universal Analysis 200 version 4.5A build 4.5.0.5 software. The measurements were performed in triplicates, at least. The mass loss at 225 °C was attributed to the total volatile content. The carvacrol content within films was calculated by subtracting total volatile content within corresponding reference films (attributed to moisture) from the total volatile content within carvacrol-containing films.

#### 2.4.3. Carvacrol Release Studies

The accelerated release of carvacrol from the films was studied by isothermal gravimetric analysis using a TGA-Q5000 system at a constant temperature of 60 °C under nitrogen atmosphere, until a constant mass was attained. 

Several methods have been reported to characterize the diffusion of active agents and other additives in polymers [41,42,43,44,45]. In this study, we express the diffusion coefficient from the initial linear slope of the fractional mass release ratio vs. $t^{1/2}$ according to Equation (1) [41,45].

(1) $$\;\frac{m_{t}}{m_{\infty }}=4{\left(\frac{Dt}{\pi l^{2}}\right)}^{1/2}\;$$

where $m_{t}$ and $m_{\infty }$ are the amounts of additive (carvacrol) released from the film at time t and at equilibrium t=∞, respectively, *D* (m^2^ s^−1^) is the diffusion coefficient and *l* is the overall film thickness. The thickness values of the specimens were determined with an accuracy of ±1 um using a hand-held micrometer (Hahn & Kolb, Stuttgart, Germany). Five readings were taken for each sample and the results were analyzed for their statistical significance using ANOVA with significance reported at the 0.05 probability level.

#### 2.4.4. Oxygen Transmission Rate 

Oxygen flux rates *J*(*t*) for the extruded multilayer films comprising LDPE/EVOH, (LDPE/HNTs)/EVOH, (LDPE/carvacrol)/EVOH, (LDPE/[HNTs/carvacrol])/EVOH and the control neat LDPE film were all determined using a MOCON (Minneapolis, MN, USA) OxTran 2/20 unit. The measurements were carried out at 1 atm pressure at 23 °C (+0.1) and a 65% relative humidity. The instrument was calibrated at 23 °C using a NIST-certified Mylar film with known oxygen transport characteristics. The oxygen transmission rate (OTR) value for each film was calculated according to Equation (2) from steady-state flux, *J*, rates,

(2) $$\;\mathrm{OTR}=J\frac{l}{\Delta p}\;$$

where, *l* is the overall film thickness and *Δp* is partial pressure difference of the oxygen across the film. The average reported P(*O_2_*) values were taken from at least two samples. The OTR values are reported as [cc·mm/(m^2^·day)].

#### 2.4.5. Antimicrobial In Vitro Assays

Micro-atmosphere diffusion antimicrobial assays: The antibacterial activity of the films was evaluated by measuring the inhibition of *Escherichia coli* (*E. coli*, ATCC 8739) growth by a modified micro-atmosphere diffusion method on LB agar [46]. *E. coli* was maintained on polystyrene beads at −80 °C and a bacterial culture was prepared by incubating one polystyrene bead in 5 mL of LB medium for 16 h at 37 °C under shaking (250 rpm). Subsequently, the culture was diluted with 0.85% *w*/*w* NaCl in distilled water to obtain a bacterial stock solution at a concentration of 10^3^ colony forming units (CFU) mL^−1^. Petri dishes containing LB agar were seeded with 0.1 mL of 10^3^ CFU mL^−1^ *E. coli* stock culture, and a film sample (with an area of ~36 cm^2^) was attached using a double-sided tape to the center of the Petri dish lid, assuring no direct contact between the film and the agar. The plates were tightly sealed with Parafilm^®^ and incubated for 12 h at 37 °C. The antibacterial potency of the films was estimated by measuring the observed inhibition zone below the films. All measurements were performed in triplicates Neat LDPE, LDPE/EVOH and (LDPE/HNTs)/EVOH films were used as reference materials.

Micro-atmosphere diffusion antifungal assay: *Alternaria alternata* (*A. alternata*; source: tomato) a phytopathogenic, clinical and food spoilage fungi was cultured on 1% potato dextrose agar (PDA: 10 g L^−1^; Bacto-agar: 15 g L^−1^ in deionized water) at 25 °C in the dark. Film efficacy was tested as follows: agar plugs (diameter = 6 mm) were removed from the edges of a 5 days old growing colony with a cork-borer. The plugs were placed at the center of a 9-cm Petri dish onto 1% PDA. Then, a film sample (6 × 6 cm; 36 cm^2^) was attached with a double-sided masking-tape to the center of the Petri dish lid (see previous section). The plates were tightly sealed with Parafilm™ and incubated inverted for 5 days at 25 °C in the dark. Neat LDPE, LDPE/EVOH and (LDPE/HNTs)/EVOH films were used as references. Following 5 days incubation, the diameter of the growing colonies was recorded, subtracting the diameter of the mycelial agar plug. Growth rate reduction was calculated as per-cent of Neat LDPE control. 

Micro-atmosphere diffusion antifungal in vivo bioassay: The efficacy of the films to extend the shelf life of products was examined using cherry tomato as a model. Fresh cultures of the decay causing molds *A. alternata* and *Rhizopus* spp. (source: tomato) were prepared by transferring mycelial fragments onto 1% PDA in 9-cm Petri dish and incubating inverted in the dark for 5 days at 25 °C. Fresh cherry tomatoes were purchased at the local grocery store. The tomatoes were surface sanitized with by thoroughly washing in tap water to remove dust followed by immersion in 10% bleach for 10 min. Then the tomatoes were rewashed with tap water and let dry on clean paper towels on the bench. Inoculation was carried out by puncturing the tomato with a sanitized scalpel, creating a 1 mm long × 1 mm deep wound. Mycelial plugs (1 × 1 mm) of *A. alternata* and of *Rhizopus* spp. (each tested separately) were placed directly on the fresh wound. The bioassays were carried out in 6-wells plate. Film samples were situated at the bottom (2 × 2 cm) and circumference (10 × 1 cm) of each well. An additional film piece (2 × 2 cm) was attached to a ’SealPlate’ cover (Excel Scientific Inc., Victorville, CA, USA), which was used to seal the entire plate at the top, creating a packaging simulation. The test system was incubated at 23 °C in the dark for 4 days. Each film sample was tested in triplicates and fungal growth was quantified as a binomial outcome (growth vs. no growth).

Micro-atmosphere diffusion antifungal in vivo bioassay: The efficacy of the films to extend the shelf life of products was examined using cherry tomato as a model. Fresh cultures of the decay causing molds *A. alternata* and *Rhizopus* spp. (source: tomato) were prepared by transferring mycelial fragments onto 1% PDA in 9-cm Petri dish and incubating inverted in the dark for 5 days at 25 °C. Fresh cherry tomatoes were purchased at the local grocery store. The tomatoes were surface sanitized with by thoroughly washing in tap water to remove dust followed by immersion in 10% bleach for 10 min. Then the tomatoes were rewashed with tap water and let dry on clean paper towels on the bench. Inoculation was carried out by puncturing the tomato with a sanitized scalpel, creating a 1 mm long × 1 mm deep wound. Mycelial plugs (1 × 1 mm) of *A. alternata* and of *Rhizopus* spp. (each tested separately) were placed directly on the fresh wound. The bioassays were carried out in 6-wells plate. Film samples were situated at the bottom (2 × 2 cm) and circumference (10 × 1 cm) of each well. An additional film piece (2 × 2 cm) was attached to a ’SealPlate’ cover (Excel Scientific Inc., Victorville, CA, USA), which was used to seal the entire plate at the top, creating a packaging simulation. The test system was incubated at 23 °C in the dark for 4 days. Each film sample was tested in triplicates and fungal growth was quantified as a binomial outcome (growth vs. no growth).

## 3. Results

### 3.1. Films Preparation and Morphology

The production of the nanocomposite multilayered films consists of several processing steps. First, carvacrol was loaded onto dry HNTs by shear mixing and ultrasonication, producing HNTs/carvacrol hybrids [23,25,26]. The latter were melt compounded with LDPE in a single screw extruder and LDPE/(HNTs/carvacrol) pellets were obtained. Finally, multilayered nanocomposite films were prepared by forced-assembly layer-multiplying coextrusion with alternating layers of the LDPE/(HNTs/carvacrol) and EVOH, termed as (LDPE/[HNTs/carvacrol])/EVOH films. The films were processed through 2, 3, 4 and 5 multipliers in a multiplication extrusion system, depicted in Figure 1, resulting in films with 9, 17, 33 and 65 layers. The total film thickness was maintained constant at 100 µm, while the weight ratio of LDPE/(HNTs/carvacrol) to EVOH was 80 to 20. Multilayered films of LDPE/EVOH, (LDPE/HNTs)/EVOH and (LDPE/carvacrol)/EVOH were produced using the same procedure. For comparison, single-layered neat LDPE, LDPE/carvacrol and LDPE/(HNTs/carvacrol) films were also produced by conventional blown extrusion. All carvacrol-containing films were transparent and continuous, with a slight yellow tint due to carvacrol presence (see Figure S1 in Supplementary Materials).

**Figure 2:**
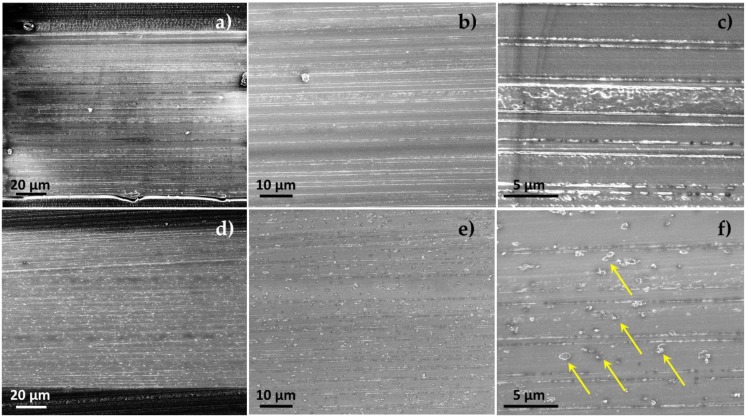
HR-SEM images of cryo-fractured cross-sections of (**a**–**c**) a 65-layer (low-density polyethylene (LDPE)/carvacrol)/ethylene vinyl alcohol copolymer (EVOH) film; and (**d**–**f**) a 65-layer (LDPE/[halloysite nanotubes (HNTs)/carvacrol])/EVOH film, at a low magnification demonstrating the entire film thickness, medium and higher magnifications. HNTs appear to be finely dispersed in the LDPE layer, marked by arrows for clarity. Note that the films were cross-sectioned in the transverse direction.

The films’ structure and morphology were studied by HR-SEM. Figure 2 presents characteristic micrographs of the cross-sectioned multilayered films. The films exhibit intact and continuous alternating layers throughout their cross-section. The calculated periodicity of the 100-µm thick 65-layer LDPE/EVOH 80/20 films is approximately 1.5 µm, consisting of alternating layers of ~1.3 µm LDPE and ~0.2 µm EVOH. Nevertheless, our microscopy studies reveal that the thickness of the individual layers is not consistent throughout the film’s cross-section, see Figure 2b,e. This behavior was also observed for all studied films, regardless of the number of layers. The HNTs are observed to be well dispersed in the LDPE matrix, and aggregates or percolating networks are not detected (see Figure 2d,e). In Figure 2f the individual HNTs are clearly observed to protrude from the film’s surface, as they are oriented in the machine direction. These results coincide well with our previous studies [23,25,26]. In general, HNTs are reported to be easily dispersed in many polymers owing to their weak cohesion, lacking strong primary interactions, mainly relying on secondary hydrogen bonds or van der Waals forces [47]. Some of the HNTs are observed to penetrate into the thin EVOH layers, however, it is impossible to determine whether it occurred during film production or was introduced during film cryo-cross sectioning.

### 3.2. Thermal Gravimetric Analysis (TGA)

As the multiplication extrusion technique process involves high exerted temperatures and long processing time (10–15 min), it may not be suitable for the processing of blends containing sensitive and bioactive ingredients. Specifically, highly volatile compounds such as carvacrol. Therefore, carvacrol retention in the multilayer films is highly important as it will determine their resulting antimicrobial functionality. The pre-processing carvacrol content in the films was 4.8% *w*/*w*, while the total carvacrol content in the films (i.e., post melt-compounding and film production) was determined by TGA and the results are summarized in Table 2. For most films, the carvacrol concentration is found to vary between 3.1–3.4% *w*/*w*, indicating that >60–70% of the initial carvacrol content is retained during the high-temperature processing steps. It should be noted that in this case, the entrapment of carvacrol within HNTs (prior to melt compounding) was not found to significantly affect its residual content within the films. Our previous studies have demonstrated that carvacrol loading into HNTs was crucial to achieve high EO content in LDPE (~70%) and prolonged antimicrobial performance [23,24]. It should be noted that in these studies the LDPE processing temperature was only 140 °C and films were prepared by compression molding and blown extrusion.

We suggest that the unique layered microstructure of the films, incorporating EVOH, which is known for its outstanding low permeability to organic vapors (including flavors and aroma compounds) [48], delays the loss of carvacrol during the extended high-temperature processing and results in a high final carvacrol content within film [36]. Thus, our results demonstrate that carvacrol can withstand the harsh conditions (high temperature and shearing) applied during the layer-multiplying coextrusion forced-assembly, resulting in uniform and transparent carvacrol-containing multilayered LDPE/EVOH films.

### 3.3. Carvacrol Release Studies

The release kinetics of the volatile antimicrobial agent from the film is crucial for determining its antimicrobial performance and potential applicability as a packaging material. Moreover, the ability to control the release kinetics is desired for tailoring the material’s properties for a specific application or condition [49]. TGA was used to characterize the carvacrol release from the different films by monitoring their weight loss over time at a constant temperature (60 °C) [26,50], and Table 3 presents the calculated effective diffusion coefficient values. The highest value of effective carvacrol diffusivity was obtained for LDPE/carvacrol films, while the addition of HNTs slows down the out-diffusion of carvacrol from the LDPE/(HNTs/carvacrol) films. This effect, was already described in our earlier work and is ascribed to the effective role of HNTs as nano-carrier for carvacrol in LDPE systems [23]. Incorporation of EVOH in the layered films has significantly lowered the effective carvacrol’s diffusivity by more than 2 orders of magnitude in comparison to LDPE/carvacrol and LDPE/(HNTs/carvacrol) films. This profound effect may be attributed to the high-barrier EVOH layered structure of the films. EVOH is well-known for its outstanding barrier properties and was shown to profoundly reduce fragrance and aroma migration [36]. The number of layers was not found to affect the effective carvacrol’s diffusivity, while the addition of HNTs induced a slight increase in diffusivity. The latter may be ascribed to the penetration of HNTs (which are 0.2 to 2 µm length) into the EVOH layers, as was observed in Figure 2, disrupting the layer integrity and enhancing carvacrol diffusion. It should be noted that in order to eliminate the plasticizing effect of water on the EVOH properties, carvacrol diffusivity was measured in dry conditions. Previous studies revealed that humidity significantly facilitates diffusion of carvacrol in EVOH systems [39,51,52].

### 3.4. Oxygen Transmission Rate 

The oxygen transmission rate (OTR) values of the multilayered films and the reference single-layers were measured and the results are summarized in Table 4. The OTR values for the carvacrol-containing multilayered LDPE/EVOH films are at least two orders of magnitude lower in comparison to those of LDPE/carvacrol and LDPE/(HNTs/carvacrol) films, demonstrating the role of EVOH as a barrier layer. In general, all carvacrol-containing films exhibit higher OTR values than their corresponding references. This effect may be attributed to the plasticizing effect of carvacrol on the polymer matrix [26,53], In the multilayered films, diffusion of carvacrol from LDPE layers to the EVOH layers, where it plasticized the EVOH matrix triggering increase in OTR. 

### 3.5. Antimicrobial Assays

**Figure 3:**
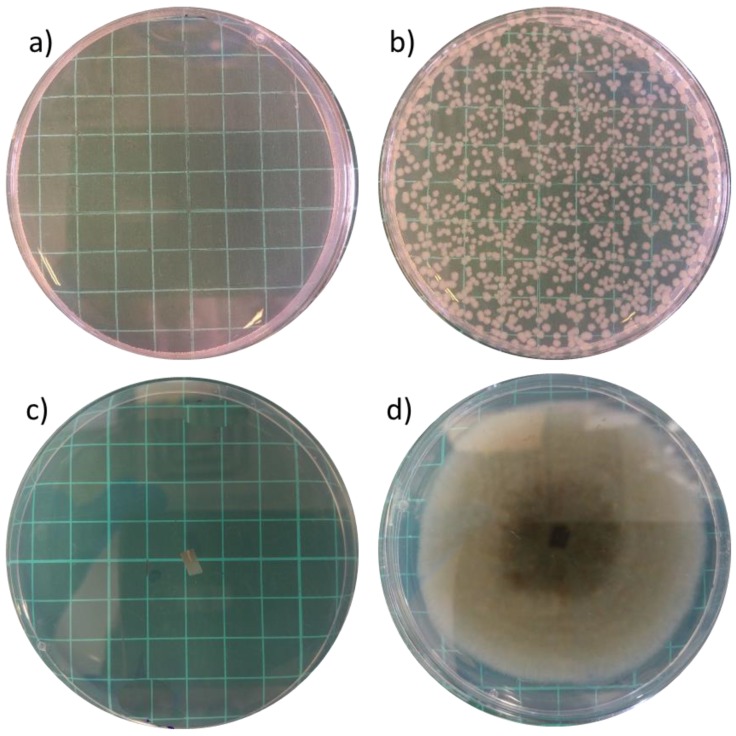
Antimicrobial and antifungal effects of the carvacrol-containing multilayered films exhibited in the micro-atmosphere diffusion *in vitro* assays, i.e., without direct contact between the studied films and the microbial cultures. (**a**) full inhibition of *E. coli* growth after incubation with a 9-layer (LDPE/[HNTs/carvacrol])/EVOH film; (**b**) unhindered *E. coli* growth after incubation with a 9-layer LDPE/EVOH film; (**c**) full inhibition of *A. alternata* development after incubation with a 9-layer (LDPE/[HNTs/carvacrol])/EVOH film; and (**d**) unhindered *A. alternata* development after incubation with a 9-layer LDPE/EVOH film. The *E. coli* were incubated with the films for 16 h at 37 °C and the *A. alternata* for 5 days at 25 °C in the dark. The results presented in (**a**,**c**) are characteristic for all carvacrol-containing films. The results presented in (**b**,**d**) are characteristic for all carvacrol-free films. All photographs were taken after the films were removed.

To evaluate the biofunctionality of the carvacrol in the films in terms of its antimicrobial activity, we studied the effect of the carvacrol-containing films on the growth of common bacterial and fungal microorganisms. The Gram-negative *Escherichia coli* (*E. coli*) was chosen as a model bacterial species as some of its strains are widely-spread foodborne pathogens. The fungus used was *Alternaria alternate* (*A. alternate*), a phytopathogen with clinical and food spoilage relevance [54]. Figure 3 depicts characteristic images of Petri dishes after the exposure of the two model microorganisms to the different films. All carvacrol-containing films were found to exhibit full inhibition of *E. coli* growth. Figure 3a shows a colony-free dish after 16-h incubation with the 9-layer (LDPE/[HNTs/carvacrol])/EVOH film, while for films without carvacrol the development of *E. coli* colonies was unhindered (see Figure 3b). All carvacrol-containing films also display a strong antifungal activity, as presented in Figure 3 and Table 5. The films completely arrest hyphal growth and sporulation of *A. alternata* as can be observed in Figure 3c. Whereas, for all carvacrol-free films no effect on fungal development is observed (see Table 5 and Figure 3d). 

**Figure 4:**
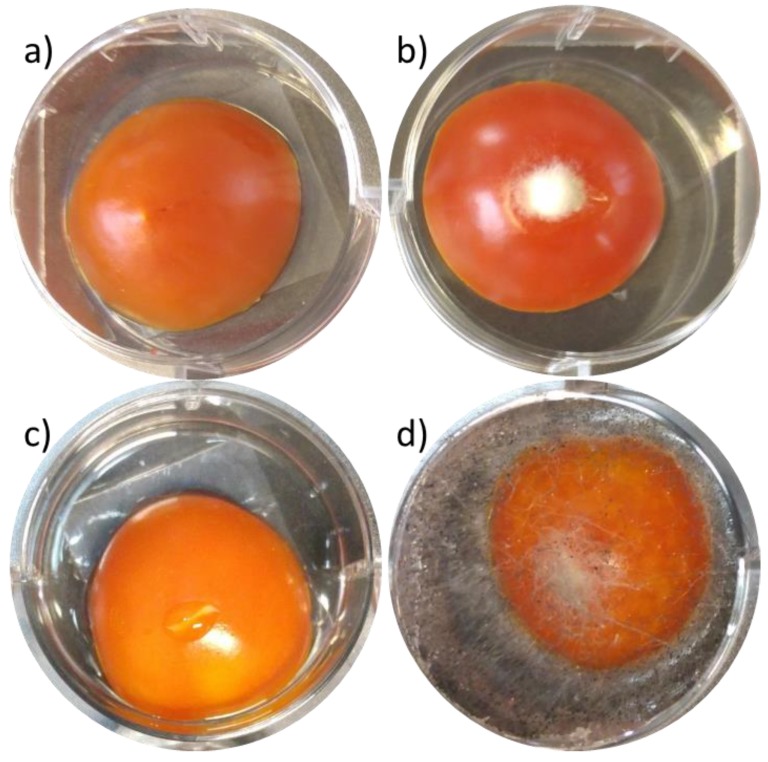
Antifungal effects of the carvacrol-containing films exhibited in the micro-atmosphere bioassays, i.e. without direct contact between the studied films and the microbial cultures using cherry tomatoes as the food model. (**a**) full inhibition of *A. alternata* development after incubation with a 9-layer (LDPE/[HNTs/carvacrol])/EVOH film; (**b**) *A. alternata* development after incubation with a 9-layer LDPE/EVOH film; (**c**) full inhibition of *Rhizopus* spp. development after incubation with a 9-layer (LDPE/[HNTs/carvacrol])/EVOH film; (**d**) *Rhizopus* spp. development after incubation with a 9-layer LDPE/EVOH film. The cherries were incubated with the films at 23 °C in the dark for 4 days. The results presented in (**a**,**c**) are characteristic for all carvacrol-containing films. The results presented in (**b**,**d**) are characteristic for all carvacrol-free films. All photographs were taken after the top and circumference films were removed.

So far, all carvacrol-containing films have demonstrated excellent *in vitro* antimicrobial activity in micro-atmosphere diffusion assay against *E. coli* and *A. alternata* on synthetic LB and PDA agar, respectively. However, EOs often exhibit inferior antimicrobial performance in real foods comparing to antimicrobial assays *in vitro*. The latter is ascribed to greater availability of nutrients in real foods comparing to synthetic growth media, which allows microorganisms to repair damage faster, and to possible interactions of EOs with various food components (e.g., fats, proteins and sugars) and other extrinsic factors (such as temperature, pH etc.) [55]. Thus, assessing antimicrobial activity of the films in real food systems is important for assessing their potential application of these films as antimicrobial packaging. Herein, the antifungal efficacy of the films was investigated using cherry tomatoes as a relevant model and *A. alternata* and *Rhizopus* spp. as model microorganisms for postharvest decay causing molds. Figure 4a,c show the outstanding antifungal activity of a 9-layer (LDPE/[HNTs/carvacrol])/EVOH film; a complete fungal growth inhibition is evident by the lack of mycelial growth and sporulation on cherry tomatoes. Table 5 summarize the *in vivo* efficacy of the studied films and the results demonstrate that carvacrol-containing films exhibit a complete fungal growth inhibition, while the reference films (no carvacrol) showed insignificant effect (see Figure 4b,d).

## 4. Conclusions

The present work shows that multilayered LDPE/EVOH films produced by forced assembly coextrusion process exhibit high carvacrol content despite the harsh processing conditions (temperature of 200 °C and long processing time). The post-processing carvacrol content was similar in all films and was not affected by the number of layers or HNTs presence at the studied concentrations. This is ascribed to the unique layered structure of the films and the barrier properties of EVOH, which makes the entrapment of carvacrol within HNTs (prior to melt compounding) not necessary for obtaining high-quality films with high carvacrol content. The multilayered carvacrol-containing films exhibit OTR and carvacrol diffusivity values that are >2 orders of magnitude lower in comparison to single-layered films (LDPE/carvacrol and LDPE/(HNTs/carvacrol)) produced by conventional cast extrusion. The (LDPE/carvacrol)/EVOH and (LDPE/[HNTs/carvacrol])/EVOH films demonstrated excellent antimicrobial efficacy against *E. coli* and *A. alternata* in *in vitro* micro-atmosphere assays and against *A. alternata* and *Rhizopus* spp. in in vivo micro-atmosphere assays using cherry tomatoes as food model. The results presented here suggest that sensitive essential oils, such as carvacrol, can be incorporated into plastic polymers constructed of tailored multiple layers without losing their antimicrobial efficacy. This is a novel technology provides the ability to customize packaging for various food products that are affected by bacteria, such as meat and fish, or by molds such as cheese, bread and fresh produce. Hence, providing food producers, farmers, and cold chain logistics personnel a versatile tool-box to overcome public health issues in situations of product abuse throughout the supply chain.

## Figures and Tables

**Table 1:** Composition of the studied multilayered films. The thickness of all films was maintained constant at 100 µm.

System	LDPE/EVOH Ratio	No. of Layers	Carvacrol Content * (*w*/*w* %)	HNTs Content (*w*/*w* %)
LDPE/EVOH	80/20	9, 17, 33, 65	0	0
(LDPE/carvacrol)/EVOH	80/20	9, 17, 33, 65	6	0
(LDPE/HNTs)/EVOH	80/20	9, 17, 33, 65	0	3
(LDPE/[HNTs/carvacrol])/EVOH	80/20	9, 17, 33, 65	6	3
Neat LDPE	N/A	1	0	0
LDPE/carvacrol	N/A	1	4	0
LDPE/(HNTs/carvacrol)	N/A	1	4	2

* Refers to initial carvacrol content in LDPE.

**Table 2:** Carvacrol content in the different films as measured by Thermal Gravimetric Analysis (TGA) (the number in parenthesis indicate the standard deviation obtained from at least three independent experiments).

Polymer Films	No. of Layers	Pre-Processing Content of Carvacrol (% *w*/*w*)	Post-Processing Content of Carvacrol by TGA (% *w/w*)
(LDPE/carvacrol)/EVOH	9	4.8	3.2 (0.1)
(LDPE/carvacrol)/EVOH	17	4.8	3.1 (0.1)
(LDPE/carvacrol)/EVOH	33	4.8	3.4 (0.1)
(LDPE/carvacrol)/EVOH	65	4.8	2.9 (0.2)
(LDPE/[HNTs/carvacrol])/EVOH	9	4.8	3.2 (0.1)
(LDPE/[HNTs/carvacrol])/EVOH	17	4.8	3.4 (0.2)
(LDPE/[HNTs/carvacrol])/EVOH	33	4.8	3.2 (0.1)
(LDPE/[HNTs/carvacrol])/EVOH	65	4.8	3.3 (0.1)

**Table 3:** Calculated effective diffusivity values for carvacrol from various films at 60 °C. Determined by fitting the mathematical model for short times diffusion-limited desorption from a polymer film surface to the release profile of carvacrol from the films measured by TGA (the number in parenthesis indicate the standard deviation obtained from at least three independent experiments).

Polymer Film	No of Layers	Carvacrol Effective Diffusivity ×10^14^, m^2^ s^−1^
(LDPE/carvacrol)/EVOH	9	1.11 ^a^ (0.19)
(LDPE/carvacrol)/EVOH	17	1.57 ^b^ (0.23)
(LDPE/carvacrol)/EVOH	33	2.22 ^c^ (0.37)
(LDPE/carvacrol)/EVOH	65	1.35 ^a,b^ (0.22)
(LDPE/[HNTs/carvacrol])/EVOH	9	2.45 ^e^ (0.08)
(LDPE/[HNTs/carvacrol])/EVOH	17	2.39 ^e,d^ (0.33)
(LDPE/[HNTs/carvacrol])/EVOH	33	2.54 ^e,d^ (0.39)
(LDPE/[HNTs/carvacrol])/EVOH	65	2.76 ^d^ (0.02)
LDPE/carvacrol	1	418 ^f^ (72)
LDPE/(HNTs/carvacrol)	1	285 ^g^ (24)

^a,b,c,d,e,f,g^—note that values with different superscripts are statistically different.

**Table 4:** Oxygen transmission rate (OTR)(*O_2_*) [cc·mm/(m^2^·day)], of the films (the number in parenthesis indicate the standard deviation obtained from at least three independent experiments).

Systems	No. of Layers			
	9	17	33	65
LDPE/EVOH	0.26 (0.03)	0.29 (0.02)	0.35 (0.02)	0.41 (0.02)
(LDPE/carvacrol)/EVOH	1.49 (0.04)	1.67 (0.04)	1.94 (0.04)	2.14 (0.06)
(LDPE/HNTs)/EVOH	0.26 (0.02)	0.28 (0.01)	0.30 (0.02)	0.33 (0.02)
(LDPE/[HNTs/carvacrol])/EVOH	1.36 (0.19)	2.31 (0.06)	2.52 (0.09)	3.24 (0.11)
LDPE/carvacrol	237.0 (6.5)			
LDPE/(HNTs/carvacrol)	254.2 (7.0)			
Neat LDPE	181.1 (5.1)			

**Table 5:** Growth reduction of *A. alternata* and *Rhizopus spp*. exposed to multilayered films. Studies were performed *in vitro* and using cherry tomatoes as a food model.

Polymer Composition	No. of Layers	*A. alternata In Vitro* Growth Reduction (%) ^x^	*A. alternata* Growth on Cherry Tomato ^y^	*Rhisopus* spp. Growth on Cherry Tomato ^y^
LDPE control 100%	1	0 ± 0	3/3	3/3
LDPE/EVOH control	9	1.1 ± 2.3	3/3	3/3
LDPE/EVOH control	17	2.9 ± 6.8	3/3	3/3
LDPE/EVOH control	33	7.2 ± 1.2	3/3	3/3
LDPE/EVOH control	65	7.9 ± 3.1	3/3	3/3
LDPE/carvacrol/EVOH	9	100 ± 0.0	0/3	0/3
LDPE/carvacrol/EVOH	17	100 ± 0.0	0/3	0/3
LDPE/carvacrol/EVOH	33	100 ± 0.0	0/3	0/3
LDPE/carvacrol/EVOH	65	100 ± 0.0	0/3	0/3
LDPE/[HNT/carvacrol]/EVOH	9	100 ± 0.0	0/3	0/3
LDPE/[HNT/carvacrol]/EVOH	17	100 ± 0.0	0/3	0/3
LDPE/[HNT/carvacrol]/EVOH	33	100 ± 0.0	0/3	0/3
LDPE/[HNT/carvacrol]/EVOH	65	100 ± 0.0	0/3	0/3

^x^ Values are mean ± standard error of the mean. ^y^ Frequency of fungal growth on cherry tomato. All tests were carried out with 3 replications per film.

## Supplementary Materials

The following are available online at http://www.mdpi.com/2073-4360/10/8/864/s1, Figure S1: Characteristic images of the multilayered films prepared by forced-assembly layer-multiplying co-extrusion a) 9-layered (LDPE/carvacrol)/EVOH; b) 65-layered (LDPE/carvacrol)/EVOH; c) 9-layered (LDPE/[HNTs/carvacrol])/EVOH; and d) 65-layered (LDPE/[HNTs/carvacrol])/EVOH.
