# Supplementary material for: Antimicrobial LDPE/EVOH Layered Films Containing Carvacrol Fabricated by Multiplication Extrusion
Source: Polymers (Basel). 2018 Aug 4;10(8):864. doi: 10.3390/polym10080864 (PMC6403741; doi:10.3390/polym10080864)
Supplement: Supplementary file 1 [file polymers-10-00864-s001.pdf]

polymers

MDPI

Supplementary Materials

Antimicrobial LDPE/EVOH layered films containing carvacrol fabricated by multiplication extrusion

Max Krepker [1], Cong Zhang [2], Nadav Nitzan [1], Ofer Prinz-Setter [1], Naama Massad-Ivanir [1], Andrew Olah[2], Eric Baer [2], and Ester Segal [1,3*]

[1] Department of Biotechnology and Food Engineering, Technion – Israel Institute of Technology, Haifa 3200003, Israel

[2] Center for Layered Polymeric Systems, Department of Macromolecular Science and Engineering, Case Western Reserve University, Cleveland, OH 44106-7202, USA

[3] The Russell Berrie Nanotechnology Institute, Technion – Israel Institute of Technology, Haifa 3200003, Israel

* Correspondence: esegal@technion.ac.il

Academic Editor: name

Received: date; Accepted: date; Published: date

Figure S1 presents characteristic images of multi-layered (LDPE/carvacrol)/EVOH and (LDPE/[HNTs/carvacrol])/EVOH films (comprising of 9 and 65 layers) fabricated by the forced-assembly layer-multiplying coextrusion technique. The films are were transparent and continuous with no apparent defects.

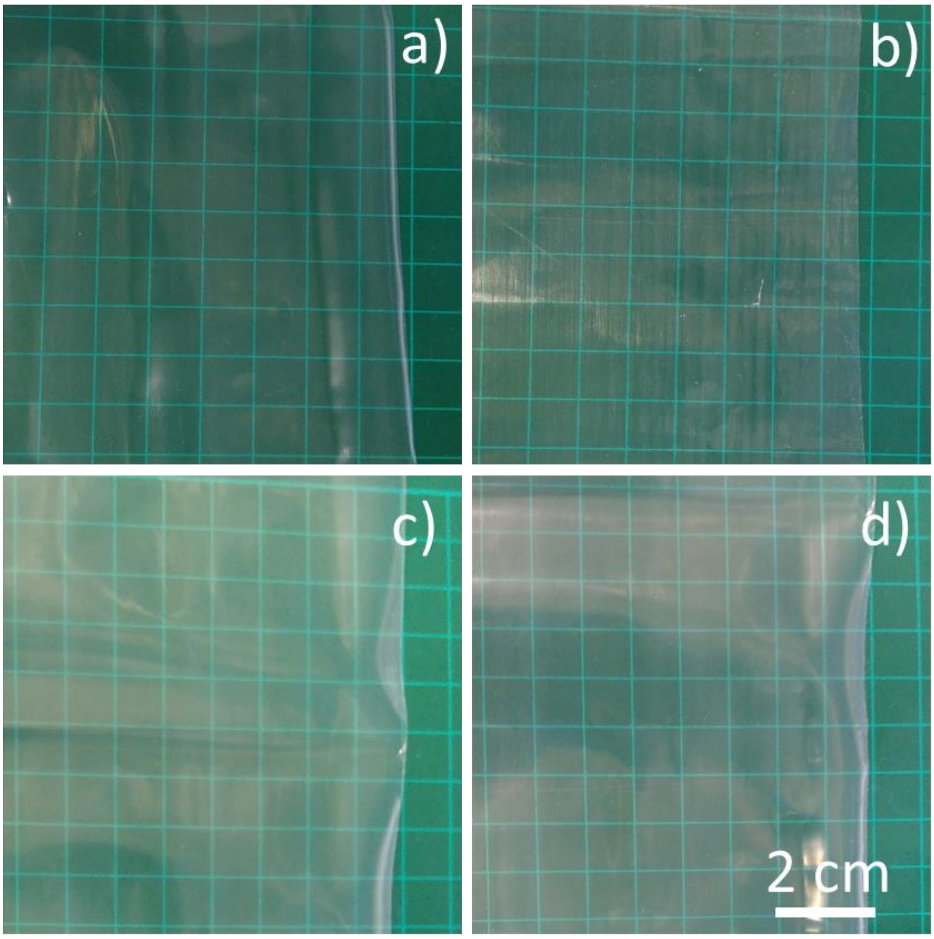

Figure S1. Characteristic images of the multilayered films prepared by forced-assembly layer-multiplying co-extrusion a) 9-layered (LDPE/carvacrol)/EVOH; b) 65-layered (LDPE/carvacrol)/EVOH; c) 9-layered (LDPE/[HNTs/carvacrol])/EVOH; and d) 65-layered (LDPE/[HNTs/carvacrol])/EVOH.

© 2017 by the authors. Submitted for possible open access publication under the terms and conditions of the Creative Commons Attribution (CC BY) license (http://creativecommons.org/licenses/by/4.0/).
